# Towards to Optimal Wavelet Denoising Scheme—A Novel Spatial and Volumetric Mapping of Wavelet-Based Biomedical Data Smoothing

**DOI:** 10.3390/s20185301

**Published:** 2020-09-16

**Authors:** Ladislav Stanke, Jan Kubicek, Dominik Vilimek, Marek Penhaker, Martin Cerny, Martin Augustynek, Nikola Slaninova, Muhammad Usman Akram

**Affiliations:** 1 Czech National e-Health Center, University Hospital Olomouc, I. P. Pavlova 185/6, 77900 Olomouc, Czech Republic; Ladislav.Stanke@fnol.cz; 2 Department of Cybernetics and Biomedical Engineering, VSB-Technical University of Ostrava, FEECS, 70800 Ostrava-Poruba, Czech Republic; dominik.vilimek@vsb.cz (D.V.); marek.penhaker@vsb.cz (M.P.); martin.cerny@vsb.cz (M.C.); martin.augustynek@vsb.cz (M.A.); nikola.slaninova@vsb.cz (N.S.); 3 Department of Computer & Software Engineering, National University of Sciences and Technology (NUST), Islamabad 44000, Pakistan; usmakram@gmail.com

**Keywords:** wavelet transformation, Daubechies wavelet, Symlet wavelet, Coiflet wavelet, spatial and volumetric modeling

## Abstract

Wavelet transformation is one of the most frequent procedures for data denoising, smoothing, decomposition, features extraction, and further related tasks. In order to perform such tasks, we need to select appropriate wavelet settings, including particular wavelet, decomposition level and other parameters, which form the wavelet transformation outputs. Selection of such parameters is a challenging area due to absence of versatile recommendation tools for suitable wavelet settings. In this paper, we propose a versatile recommendation system for prediction of suitable wavelet selection for data smoothing. The proposed system is aimed to generate spatial response matrix for selected wavelets and the decomposition levels. Such response enables the mapping of selected evaluation parameters, determining the efficacy of wavelet settings. The proposed system also enables tracking the dynamical noise influence in the context of Wavelet efficacy by using volumetric response. We provide testing on computed tomography (CT) and magnetic resonance (MR) image data and EMG signals mostly of musculoskeletal system to objectivise system usability for clinical data processing. The experimental testing is done by using evaluation parameters such is MSE (Mean Squared Error), ED (Euclidean distance) and Corr (Correlation index). We also provide the statistical analysis of the results based on Mann-Whitney test, which points out on statistically significant differences for individual Wavelets for the data corrupted with Salt and Pepper and Gaussian noise.

## 1. Introduction

As far as it is commonly known, as the result of the physical limitations of most medical systems, signals and images have the tendency to manifest some random noise within signal and image acquisition [1]. In a general way, such additive noise is perceived as a data distortion, which may significantly deteriorate the process of data observation, analysis and feature extraction [2,3,4]. In the present time, we are overcrowded by plenty acquiring medical devices, intended for data acquisition, where the resulting signals and images may be affected by different types of the noise. Such noise sources have the potential to significantly deteriorate quality of clinical information [5,6,7]. Therefore, the smoothing methods are gaining a significant importance in the computer aided analysis. Among others, Additive White Gaussian Noise (AWGN), impulse noise (also called Salt and Pepper), quantisation noise, Poisson noise, and speckle noise are frequently discussed in the recent literature [1,8,9]. Considering different nature of individual types of the noise, we can deduce that individual smoothing methods may be differently sensitive and robust to each of the noise type. This hypothesis deals with the fact that there is not unified method, with unified settings which would perform the data smoothing, corrupted by different noise manifestation with the same effectivity [10,11].

By using the basic formulation of digital signal, we use the model, representing a sequence (matrix), corresponding with individual discrete samples (Equation (1)). In the case of the digital image, this form is encoded as a matrix, containing gray-level or colour pixels (voxels) [12,13,14]. These elements represent image intensities. In the case of the gray scale (so called monochromatic) images, the form is represented as 2D image: (r,u(r)), where u(r) represents the image intensity in the coordinates: r=[x,y]. In the case of the colour image, u(r) assumes a triplet of the intensity values individual chrominance channel: red, green and blue [15,16]. When assuming the noise presence, we need to incorporate into formulation of the signal function noise model, which is denoted as n(r) in Equation (1).

(1) f(r)=u(r)+n(r),⊂R,R⊂Z.

This noise can be additive or multiplicative in nature. After formulation of the noise model, it should be noted that the additive noise is firmly bounded with the original (native) information, which is transmitted from the human body by the form of signals or images [17,18]. Therefore, the resulting magnitude of the signal element f(r) is composed as from the native clinically important information, as from the noise sample. Thus, these components cannot be completely separated. Based on this fact, by utilizing the image smoothing we perform a prediction of native signal u(r) by suppressing n(r). Based on this principle, we always at least partially suppress the native clinical information, and perceive the additive noise. For this reason, we usually search for a compromise between non-distorting clinical information and in the same time elimination as much noise level as possible [19,20,21].

There are various reasons for incorporating the data smoothing procedures. Standardly, these methods are applied prior the methods for identification and classification of clinical information. In the case of the signals, we commonly need to detect (identify) the signal trend to analyse evolution of the biologic signals over the time. The signal noise often causes a signal distortion in the form of glitches and oscillations [21,22]. Such phenomena have the tendency to affect the signal morphology and influence signal trend.

In this paper, we analyse the performance, including effectivity and robustness of the Wavelet transformation by a novel metric, utilizing the spatial and volumetric modelling of objective parameters. Among others smoothing procedures, Wavelet transformation plays a crucial role. In the contrast with many other methods, Wavelet transformation allows for the data decomposition on various scales. Such procedure makes a bank of the filters instead of single filtering procedures. The leading benefit of the Wavelet transformation is a large selection from mother’s Wavelets and their families. These facts predetermine various using of this method for multiple purpose. We bring a novel method, utilizing construction of spatial and volumetric matrixes, allow for mapping Wavelet behaviour within Wavelet families and individual scales. Mapping of objective parameters, including correlation coefficient, Mean Squared Error (MSE) and Euclidean distance (ED) enables analysis of distribution of the Wavelet behaviour with various settings. We publish an extensive testing of performance distribution over electromyography (EMG) signals and various imaging systems (computed tomography (CT) and magnetic resonance (MR)) to report a comprehensive comparative analysis of the Wavelet performance in different data processing. In the further step, we aimed on the study of the dynamic noise influence on the Wavelet behaviour. Such extensive analysis points out on the Wavelet behaviour upon dynamical features of the noise and types of the noise over the time. The structure of the paper is follows. Section 2 deals with the recent methods and trends of the procedures for biomedical data smoothing. In the Section 3, we publish datasets of 1D signals and 2D images, which are used for the testing and evaluation of the proposed methods. Section 4 deals with a proposal and realization of spatial and volumetric modelling of the Wavelet response. Section 5 deals with analysis of the results. We mainly analyse the spatial and volumetric characteristics for 1D and 2D data, which are corrupted by the additive Salt and Pepper and Gaussian noise. Part of this analysis is statistical testing of the significance of the distributions of Wavelet response parameters.

## 2. Recent Work

In the recent literature, there are plenty contributions, reporting image-based smoothing (filtering) for medical image data enhancement, typically for medical signals and images. Generally, the data smoothing are mostly performed either in spatial or frequency domain [23]. The filters, utilizing the pixel’s distribution have become mainly interesting for the research community. Here, we recognize the local filters, considering a certain finite representative pixel neighbourhood. Based on the local statistical features they are capable of modifying the pixels values [24,25]. Such principle is utilized in average, median, Gaussian filter or bilateral filter. One of the frequently used filters is Wiener filter [26]. This filter do not employ mean of the neighbourhood pixels, but involve linear estimation of desired signal sequence based on the another related sequences [27,28]. Such filters often blur edges as a negative side effect of the filtering, conversely they are able to effectively suppress local noise. Such local window may be argument of limitations of these filters due to approximation only local features of the image spatial area [29,30]. In the contrast of this principle, non-local filtering is often applied in the medical image processing [31,32]. In this category, we point out on well-known Non Local Mean Filter (NLM filter). The advantage of NLM filter is the fact that all the surrounding pixels may affect the representative pixels, without restriction of the weighting function [33,34]. This type of the filtering incorporates two features: besides the intensity information NLM filter works with the distance function between representative pixel and others [35,36,37,38].

The further popular techniques for image smoothing are the methods based on the random fields. These methods work with the image intensities as dependence of adjacent intensity values [39]. These methods are based on the observation that the global representation of the medical image can be estimated from its local physical structures based on conditional probability distribution function. Such popular principle is called Markov random field (MRF) [40,41].

Another challenging field in the image filtering is the redundant and sparse representation. These concepts of sparsity and redundancy have been extensively applicable with using of various multi-scale and directional transforms, such is curvelets, ridgelets, brushlets, and directionlet [42]. One of the popular approach that introduce sparsity and redundancy for image representation is the sparse representation based on the dictionary learning. As typical member of this category is BLS-GSM (Bayes Least Square Gaussian Scale Mixture). This approach translates the image denoising problem into an inverse problem with the use of Bayesian minimisation [42,43].

The last important section of the image smoothing is the category transform domain filtering. When comparing with the spatial domain, transform domain methods take advantage of the sparsity features [44,45]. For instance, the signals or images may be represented by a shorter sequence of non-zero coefficients, such is the Fourier coefficients. In this category, we state Fourier transform (FT), Fast Fourier transform (FFT), discrete cosine transform (DCT), curvelets, ridgelets, ripplet, contourlet, wedgelets, directionlet, shearlet, and mainly Wavelet transformation [46,47,48]. Among other domain filtering methods, Wavelet transformation plays a significantly important role [30,49]. Besides a multiple using of Wavelet transform, this method allows various settings, including scaling (filter bank) and wide possibilities of Wavelet families and individual wavelets for various applications to various medical data [50,51,52,53,54,55]. These extensive settings create challenging issues. Firstly, the behaviour of Wavelet transformation among individual settings, application, effectivity and robustness of Wavelet transform for various image sources, including the study of the feasibility of particular wavelets for various image structures (CT, MR, ultrasound, and others), and lastly the prediction of Wavelet dynamical behaviour when the noise with the dynamic intensity effect is presented.

## 3. Data Analysis

**Figure 1:**
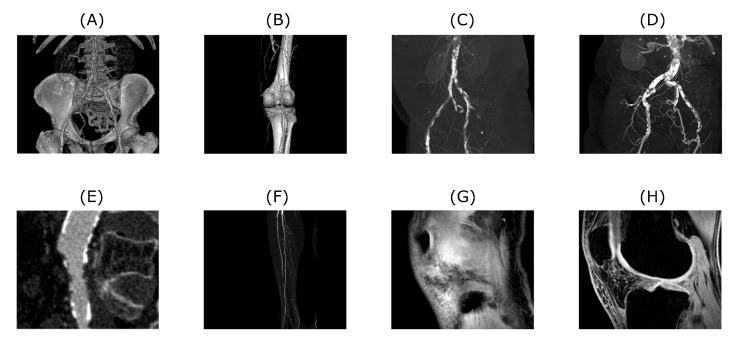
Examples of CT (**A**–**F**) and MRI (**G** and **H**) scans used to test the proposed algorithm. Tested images are showing various level of magnification and amount of detail. Also, native noise levels contained within these images are different. Images (**A**–**F**) are depicting blood vessel calcification in different details and perspectives, whereas images (**G**–**H**) are showing successive slices of knee cartilage.

To properly analyze the performance of the approach described in the next chapter, a bank of input test data needs to be prepared and described first. In our research, we are primarily focused on the analysis of the 1D and 2D image data mostly of the musculoskeletal system. These input test data were selected to benchmark the proposed algorithm. Datasets are mostly related to the human’s musculoskeletal system, however, algorithm’s application is not limited to this specific field. As for 1D signals we used EMG measurements of hand movements from publicly available data source [56]. The algorithm’s performance of 2D datasets was tested on CT and MRI scans. CT scans are showing blood vessel calcification in the area of lower limbs. There are several levels of detail magnification so images with different properties like spatial frequency could be tested. MRI dataset is consisting of successive slices showing knee cartilage in order to test various spatial frequency images. Examples of various musculoskeletal images used for the analysis are depicted in Figure 1.

Although, the selected 1D signals and 2D images (mostly of musculoskeletal apparatus) are from the real clinical measurements and basically consist of some level of native noise (commonly Rician distribution of noise in MRI images etc.), they are taken as ideal ones and additional level of artificial noise is subsequently applied to them for testing the proposed algorithm noise filtration performance. Obviously, the algorithm can be tested with artificially generated signals as well. This is especially easy to do with ECG signal generators, both hardware and software ones. Nevertheless, it was decided to use the real data for all presented tests.

As it was mentioned earlier there are several types of noise contained within the measured 1D signal or 2D image. To test the algorithm’s performance following types of noise are applied to the selected datasets. Namely, additive white Gaussian noise and impulse noise for 1D signals and for the 2D images the same types of noise also with added speckle noise. Besides already mentioned additive white Gaussian noise, it is also speckle noise, Poisson noise and impulse noise. Impulse noise applied in the 1D case is added via own part of the code. In both presented versions of the algorithm—1D and 2D—the applied amount of the noise level is being varied with use of a for loop to simulate the effect of dynamical noise intensity. As variables were chosen mean of the Gaussian noise and noise density of salt and pepper noise. During the applied for loop both values are being steadily increased. For 2D signal also a speckle noise is being added, however, in contrast with previously mentioned types, the speckle noise is set to be constant across the for loop. Speckle noise is described as multiplicative noise using the Equation (2):

(2) J=I+n∗I,

where *n* is uniformly distributed random noise with mean 0 and variance 0.05. These default parameters of speckle noise were kept also for the presented algorithm tests.

## 4. Materials and Methods

In this section, we describe the construction of 2D spatial and 4D response of Wavelet families. These characteristics reflect the Wavelet response in the form either spatial or volumetric distribution of evaluating parameters. Spatial characteristics reflect the Wavelet response for a single-intensity noise and volumetric characteristics contrarily allow for tracking of dynamical effect of the Wavelet effectivity upon the whole scale of noise intensity.

Presented algorithms (for use with 1D and 2D input) exploits the advantages of Wavelet transforms over Fourier transform. Wavelet transform is defined as a degree of correlation W(a,b) between the signal s(x) and the analysing wavelet $\psi_{a,b}$ given by the relation (3):

(3) $$W\left(a,b\right)=\int_{-\infty }^{\infty }s\left(x\right)\psi_{a,b}\left(x\right)dx,$$

where analyzing wavelet $\psi_{a,b}$ is described by the Equation (4):

(4) $$\psi_{a,b}=\frac{1}{\sqrt{a}}\cdot \psi \left(\frac{x-b}{a}\right),$$

in which *a* and *b* represents scaling and shifting factor, respectively. The function ψ(x) is called mother wavelet and unlike in Fourier transform the mother wavelets can be given by other functions different than the goniometric. During the history many mother wavelet types were proposed. In the context of the presented paper three distinctive families are exploited—Daubechies, Symlet and Coiflet. They are all representations of Discrete Wavelet Transform (DWT). As the highest available order N of Coiflet mother wavelet is 5, it is decided that others of the used mother wavelets are also tested up to this order.

### 4.1. Data Preparation

**Figure 2:**
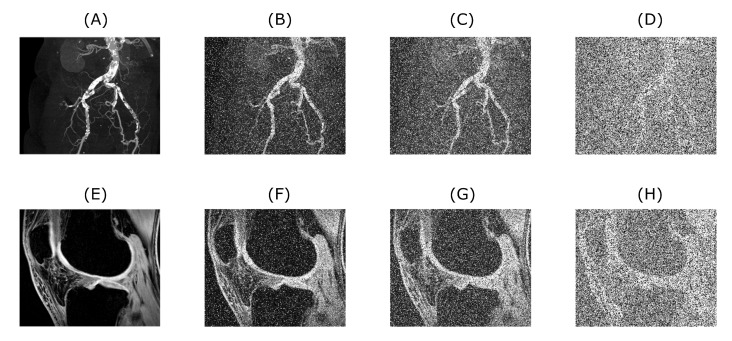
Upper row is showing a CT scan of blood vessel calcification with the native level of noise (**A**) used as an input for algorithm testing. Images (**B**–**D**) are showing the original image with added artificial noise for 0.1, 0.2 and 0.5 level (in fact the noise consists of a sum of three noise types, in which the number means a mean value for additive white Gaussian noise and density for salt and pepper noise, speckle noise is kept on a constant default value used in MATLAB), respectively. Lower row shows the example of MRI scan of knee cartilage (**E** used as an input), again with different consecutive noise levels of corresponding magnitude (**F**–**H**).

**Figure 3:**
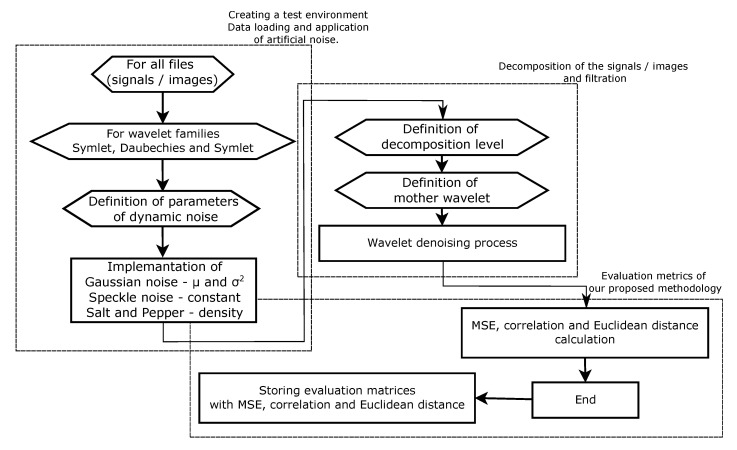
A scheme of a proposed algorithm for 1D and 2D signal (e.g., EMG, CT or MRI) denoising.

The wavelet filter testing was performed in MatLab (MathWorks, Natick, MA, USA) and conducted as follows. Firstly, we prepared 1D and 2D banks of samples. These consisted of EMG signal measurements, MRI and CT scans. Signals and images were chosen, representing different spatial frequencies and levels of native noise. These signals or images were loaded into a multiarray structure so the algorithm can be tested on each signal or image from the prepared bank of 1D and 2D inputs. The first for loop simply selected one signal or image after the other. A level below this for loop is a for loop that varies the level of added artificial noise (as demonstrated in Figure 2). These procedures are illustrated within the two algorithm schemes for both cases of 1D and 2D input as shown in Figure 3, described in natural language below the figures as well.

1D signals were degraded by noise in the part of the code which also adds an impulse type of noise. The parameters over which the for loop is iterated is the signal to noise ratio of the awgn (Add white Gaussian noise to signal) function and the variance of the impulse noise. Both values were increased with each iteration of the for loop. Both types of noise were added simultaneously. For 2D signals, we implemented procedures for adding additive white Gaussian, impulse and speckle noise. With the for loop, the mean value of the additive white Gaussian noise was iterated together with the density of the impulse (Salt and Pepper) noise, while the speckle noise was held constant.

### 4.2. A Quantitative Measures of Wavelet Effectivity

Finally, for each noise level, there are two for loops governing the order of the mother wavelet and the level of decomposition. For the chosen mother wavelet—Daubechies, Symlet or Coiflet—a denoising was performed in the MatLab’s Wavelet Toolbox. The filtered image was adjusted with the use of the image adjust function (linear intensity transformation) contained within the Image Processing Toolbox. It was found out via testing on the available images that the optimum default value for the low intensity in the parameters of the image adjust function lies between 0.4 and 0.6, which gives the best results for the majority of the images. After this step, three distinctive images are available—the original input, an image that has an added artificial noise over the original one and finally the denoised image. Since the comparison between them can be conducted with the use of several approaches, a benchmark of the denoising is performed. Namely a MSE, Corr and ED or norm, in the case of 2D matrices described as:

(5) $$MSE=\frac{1}{mn}\Sigma_{i=1}^{m}\Sigma_{j=1}^{n}{\left[X_{ij}-Y_{ij}\right]}^{2},$$

(6) $$Corr2D=\frac{\Sigma_{i}\Sigma_{j}\left(X_{ij}-\bar{X}\right)\cdot \left(Y_{ij}-\bar{Y}\right)}{\sqrt{\left(\Sigma_{i}\Sigma_{j}{\left(X_{ij}-\bar{X}\right)}^{2}\right)\cdot (\Sigma_{i}\Sigma_{j}\left(X_{ij}{\left(Y_{ij}-\bar{Y}\right)}^{2}\right)}}.$$

(7) $$Euclidean\;distance=\sqrt[{2}]{\Sigma_{i=1}^{m}\Sigma_{j=1}^{n}{\left[X_{ij}-Y_{ij}\right]}^{2}},$$

where $X_{ij}$ and $Y_{ij}$ are elements of the two matrices that are being compared and $\bar{X}$ and $\bar{Y}$ their mean values. These functions are compared between the denoised and the original input image, which is perceived as a gold standard. There is the following relation between the Peak Signal-to-Noise Ration (PSNR) and MSE:

(8) $$PSNR=10\log_{10}\left(\frac{peakvalue^{2}}{MSE}\right).$$

For each combination of wavelet transform settings the above mentioned denoise performance metrics are calculated.

**Figure 4:**
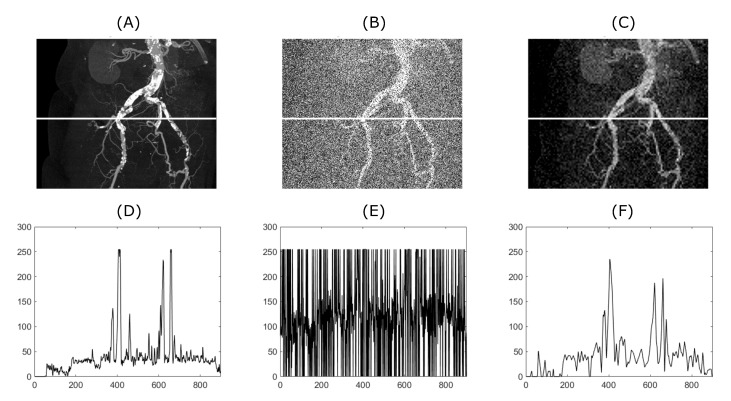
From (**A**) to (**C**), the upper row shows the original image, the image with added artificial noise and the image denoised by the described algorithm. As an input image, a CT scan shown in Figure 1 and Figure 2 was used. The lower row (**D**–**F**) shows plots corresponding to the white line drawn in the images depicted in the upper row.

**Figure 5:**
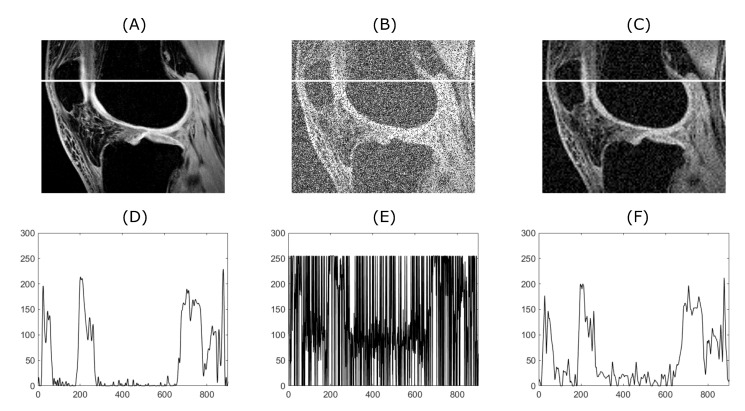
From (**A**) to (**C**), the upper row shows the original image, the image with added artificial noise and the image denoised by the described algorithm. As an input image, an MRI scan shown in Figure 1 and Figure 2 was used. The lower row (**D**–**E**) shows plots corresponding to the white line drawn in the images depicted in the upper row.

Figure 4 and Figure 5 demonstrate the relationship between the original input image, the image with added artificial noise and the denoised image. In these specific cases, both variables (mean value of AWGN and density of salt and pepper noise) governing the amount of an artificial noise were set to 0.35. Low-in value of the image adjust function was set to 0.45 and 0.4, for CT and MRI image, respectively. As two specific images are chosen from the range of the batch dataset, the image adjust function parameter is tweaked to achieve better performance. Algorithms 1 and 2 below describing procedure for solving a problem, based on conducting a sequence of a denoising for 1D a 2D signals.
**Algorithm 1** 1D algorithm natural language description**Step 1**: load *.mat file with EMG signals from publicly available database**Step 2**: For wavelet family Symlet, Dabeuchies and Coiflet test the signal denoising**Step 3**: For snr values 0.05:0.05:10; generate artificial noise as a sum of additive white Gaussian noise(awgn(signal,snr,‘measured’)) and impulse noise with variance equal to 1/snr**Step 4**: For decomposition level 1:1:5**Step 5**: For wavelet type 1:1:5 (e.g., Sym1, …, Sym5)**Step 6**: Denoise the signal with use of wavelet (noise reduction of the signal based on thresholding ofwavelet coefficients using a global threshold proposed by Donoho et al. [57].)**Step 7**: Calculation of MSE—comparison of input and denoised signal**Step 8**: Calculation of correlation level—comparison of input and denoised signal**Step 9**: Calculation of Euclidean distance between the input and denoised signal**Step 10**: Iterate to next wavelet type**Step 11**: End for**Step 12**: Iterate to next decomposition level**Step 13**: End for**Step 14**: Iterate to noise level**Step 15**: End for**Step 16**: Iterate to next wavelet family**Step 17**: End for**Step 18**: Storing of evaluation matrices for MSE, correlation level and Euclidean distance
**Algorithm 2** 2D algorithm natural language description**Step 1**:Scan folder for available image files and create multiarray structure of images**Step 2**: For wavelet family Symlet, Dabeuchies and Coiflet test the image denoising**Step 3**: For var values 0, 0.1, 0.2, 0.3, 0.4, 0.5, 0.6, 0.7, 0.8, 0.9, 1; generate artificial noise as a sum ofadditive white Gaussian noise (imnoise(Input,’gaussian’,var)), speckle noise (imnoise(Input,’speckle’);and salt and pepper noise (imnoise(Input,’salt & pepper’,var); with AWGN mean value equal to var,salt and paper noise density equal to var and speckle noise equal to the MATLAB‘s constant defaultand add it to all loaded input images**Step 4**: For decomposition level 1:1:5**Step 5**: For wavelet type 1:1:5 (e.g., Sym1, …, Sym5)**Step 6**: Denoise the image with use of wavelet (noise reduction of the signal based on thresholding ofwavelet coefficients using a global threshold proposed by Donoho et al. [57].)**Step 7**: Calculation of MSE—comparison of input and denoised image**Step 8**: Calculation of correlation level—comparison of input and denoised image**Step 9**: Calculation of Euclidean distance between the input and denoised image**Step 10**: Iterate to next wavelet type**Step 11**: End for**Step 12**: Iterate to next decomposition level**Step 13**: End for**Step 14**: Iterate to noise level**Step 15**: End for**Step 16**: Iterate to next wavelet family**Step 17**: End for**Step 18**: Storing of evaluation matrices for MSE, correlation level and Euclidean distance

### 4.3. Spatial and Volumetric Modelling of Wavelet Response

In this section, we closely introduce the proposed model for spatial (2D) and volumetric (4D) evaluation of Wavelet settings effectivity for data smoothing. As we stated earlier, we propose a method which aims for the mapping of the Wavelet response distribution for a specific range of the mother’s wavelets and the decomposition levels. Such an approach has the potential to provide quantitative visualization of comparison effectivity of various Wavelet settings. This approach brings novel benefits for the evaluation of Wavelet effectivity via simultaneous analysis of different Wavelet settings as for the images, corrupted by noise with steady intensity, as well as the noise with dynamic intensity. This approach allows for tracking Wavelet effectivity over dynamic intensity of image noise, besides these quantitative distribution characteristics.

**Figure 6:**
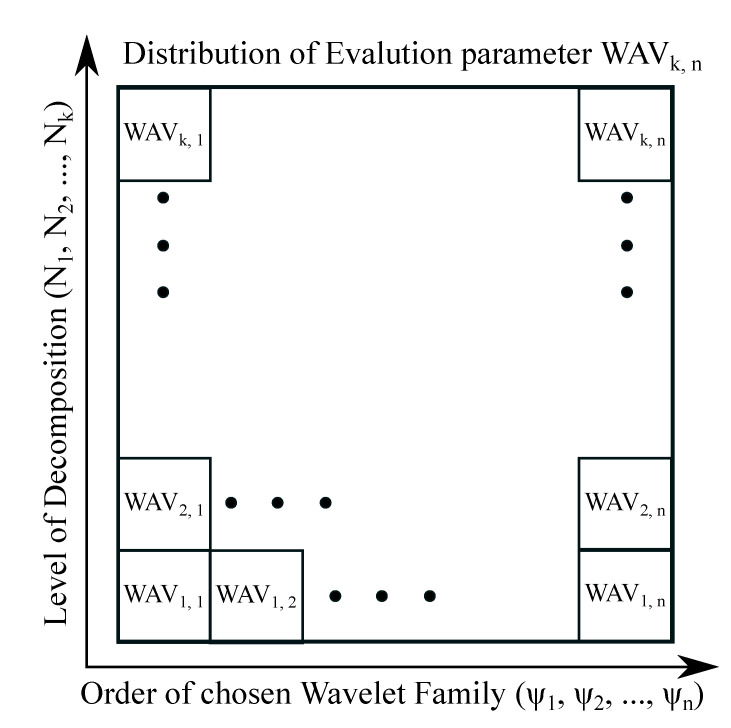
A general Scheme of spatial modelling for 2D distribution of Wavelet response.

Regarding single noise intensity, we propose a 2D characteristic, which is called the spatial 2D Wavelet map (Figure 6). This map represents a distribution of the Wavelet effectivity measured based on the quality parameters like is MSE, Euclidean norm and other parameters stated in the Section 4.1. This distribution of respective parameter provides a visualization in artificial color coding of effectivity of respective Wavelet family and various decomposition levels (range is specified by user). This method allows for effectively evaluating individual Wavelets among each other and track their effectivity as a recommendation tool for selection of the most suitable Wavelet. The spatial modelling scheme (Figure 6) contains a distribution of elements $WAV_{k,n}$, where n denotes the Wavelet order and k is a level of decomposition. This scheme generates a spatial distribution matrix *k* multiplied by *n* giving the Wavelet response for particular Wavelet family. Note that $WAV_{k,n}$ stands for respective evaluation parameter highlighting a level of difference (similarity) between the respective Wavelet response and gold standard. Based on the principle of spatial distribution mapping, such an approach can be used for single noise level evaluation.

**Figure 7:**
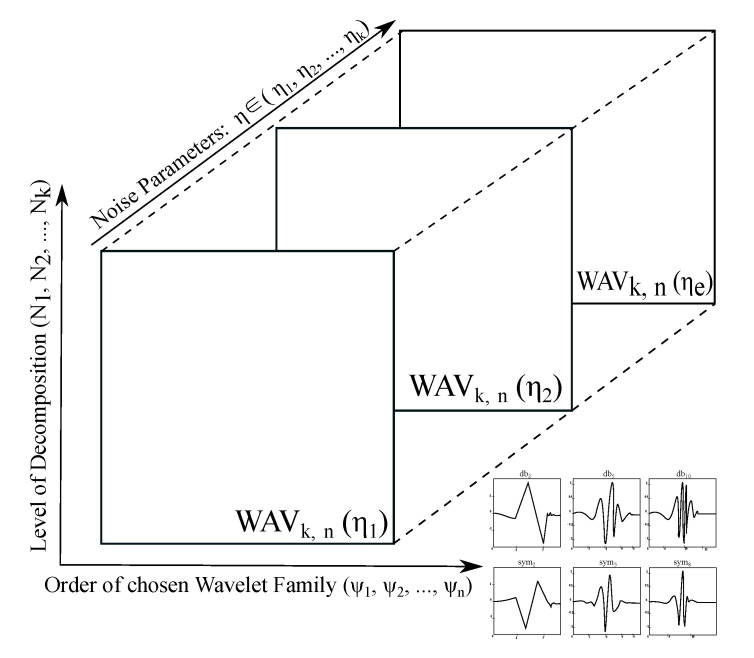
A general scheme of volumetric (4D) modelling for 2D distribution of Wavelet response.

The spatial modelling allows only for analysis of the multiple wavelet response for single noise intensity. This fact may be complication in the case of robustness of Wavelet analysis. In this context, we understand the Wavelet robustness as a dynamical evolution of change effectivity when the dynamic intensity noise is present. Therefore, in our analysis, we also propose an extended version of the spatial modelling in the form of the volumetric (4D) modelling (Figure 7) of the Wavelet response. This model allows for the dynamic features progress, depending on the noise intensity. As a result of this approach, we can extract a spatial distribution for any noise level, or track effectivity within the noise influence. The scheme in Figure 7 represent a general Wavelet response with using evaluation parameter *WAV_k,n_*, depending on the dynamical noise, represented by the parameter *η*.

## 5. Results

**Figure 8:**
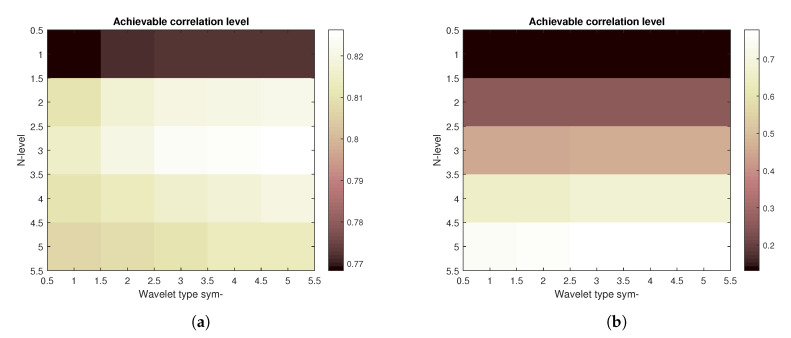
Achievable correlation level for different combinations of Symlet mother wavelet order and decomposition level for lower (**a**) and higher (**b**) amount of added artificial noise

Firstly, the construction of the results based on the spatial modelling is shown in Figure 8 is illustrating the result of achievable correlation level for two specific amounts of noise. Left part of the figure is showing correlation levels for the image with the noise parameters set as follows—the Gaussian mean is 0.1 and Salt and pepper density is also 0.1, both noise types are being summed together. The right part of the picture shows the corresponding matrix for Gaussian mean of 1 and Salt and pepper density also set to 1, both noise types are again summed together.

**Figure 9:**
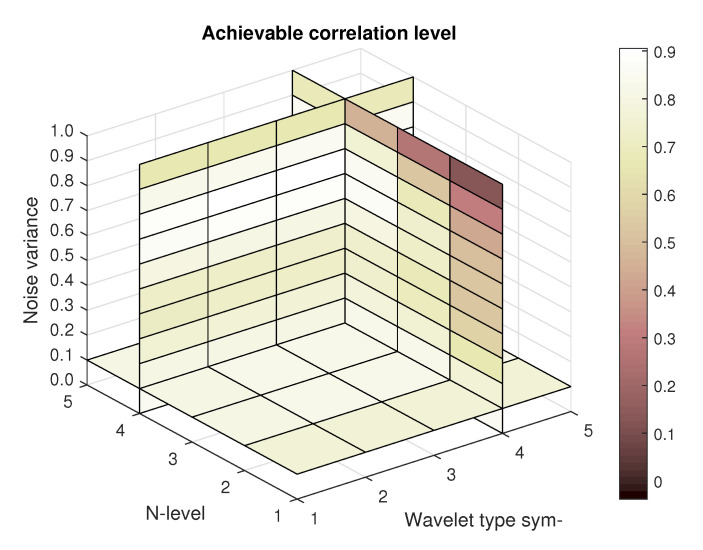
Construction of a 4D matrix with achievable correlation level for different combinations of Symlet mother wavelet order and decomposition level for various amounts of added artificial noise as a composite of matrices shown as examples in Figure 8.

In the next step, 4D matrices containing the information on achievable correlation, mean-squared error and Euclidean distance are created as a composite of the 3D matrices created for each noise level (example of such 4D matrix with the achievable correlation level is shown in Figure 9). The element composition of each 4D matrix is as follows: decomposition level, wavelet order, noise variance, image No.). By the noise variance it is meant magnitude of either white noise and impulse noise, while in the first case the noise variance is in the sense of mean value, whereas in the latter one in the sense of density. Both noise types are summed up and being added to the original input image simultaneously. As a result for each setting of noise parameters values of the highest achievable correlation, the lowest mean-squared error and the shortest Euclidean distance can be identified. Correspondingly to those values, settings for wavelet transform denoising can be assigned, therefore identifying ideal settings for achieve maximum denoising effect.

**Figure 10:**
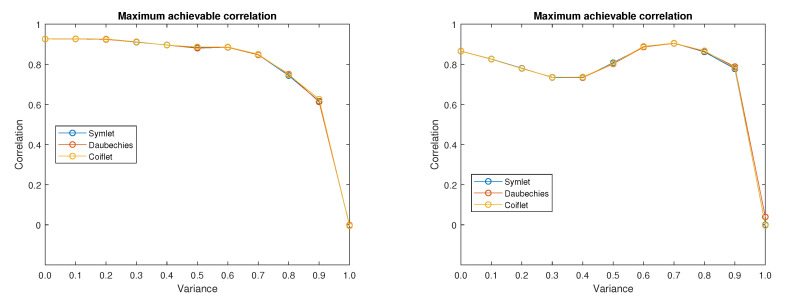
Maximum achievable correlation levels with use of three mother wavelet families for two different input images.

Figure 10 shows the plots of maximum achievable correlations for all three analysed wavelet families regardless of the specific combination of possible wavelet order and decomposition level. Results corresponds to input images (left plot) and 5 (right plot) from Figure 1. From the results it is obvious that for the chosen input images the influence of the used wavelet family is rather low. This correspond also to the results shown in Reference [58], where it is mentioned that the Symlet wavelet family outperforms the other families, even though the plotted results do not show a significant difference between the Daubechies and Symlet wavelet families shown in this paper. The Coiflet family is not included in the mentioned comparison. In the tested MRI image database, the mean difference from achievable correlation values between input and denoised images is 0.0033 for the Coiflet family, −0.0024 for Daubechies family and −0.0009 for Symlet family. For the tested CT images the worst result is also achieved with the Coiflet family with mean difference from achievable correlation is 0.0021, whereas for both Daubechies and Symlet the result is equal to −0.0010. An important variable is decomposition level and the order of the chosen wavelet family. Typically for the images with lower amount of noise level the correlation is increasing with the higher decomposition level and after reach of its maximum value the achievable correlation starts to decrease. The higher the amount of noise, the higher decomposition level is desirable.

From Figure 10 it is obvious that there exists some noise level threshold value from which the maximum achievable correlation drops fast to zero. Before reaching this threshold value the maximum achievable correlation levels are higher than 60%. It is likely that the maximum achievable correlation would be even better if the low-in value of the increased contrast of the output image is tuned specifically for each tested image.

**Figure 11:**
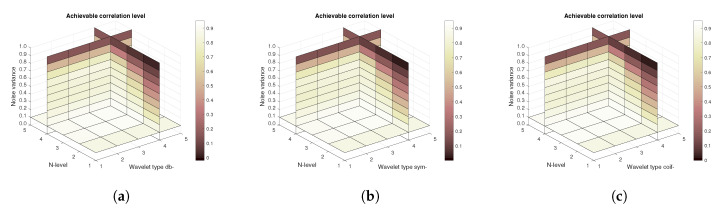
Mean achievable correlation levels for 60 MRI images with use of three mother wavelet families—from left Daubechies (**a**), Symlet (**b**) and Coiflet (**c**).

**Figure 12:**
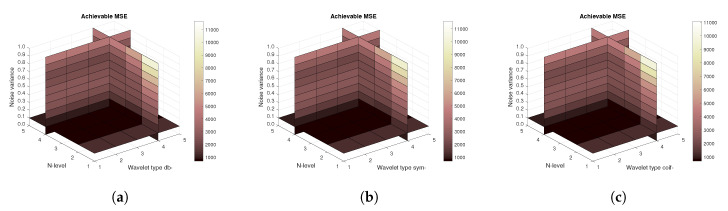
Mean achievable MSE values for 60 MRI images with use of three mother wavelet families—from left Daubechies (**a**), Symlet (**b**) and Coiflet (**c**).

**Figure 13:**
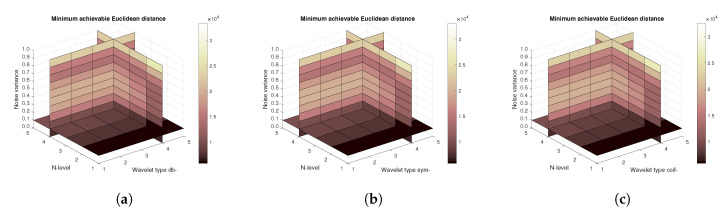
Mean achievable Euclidean distances for 60 MRI images with use of three mother wavelet families—from left Daubechies (**a**), Symlet (**b**) and Coiflet (**c**).

Figure 11, Figure 12 and Figure 13 show the results that are achieved with the database of 60 MRI scans—consecutive scans of knee cartilage. Firstly, the mean achievable correlations levels are shown (Figure 11), then the mean achievable MSE (Figure 12) values, and finally the mean Euclidean distance (Figure 13). Differences among the Daubechies, Symlet and Coiflet families is negligible. In these cases, both AWGN and Salt and pepper noise were present in the tested images.

**Figure 14:**
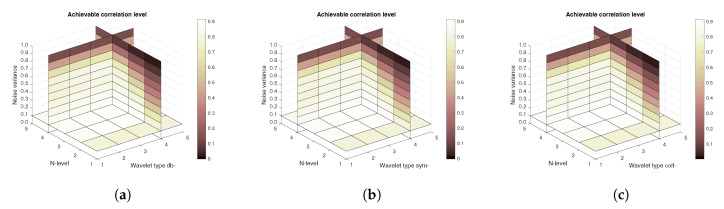
Mean achievable correlation levels for 15 CT images from 5 patients with use of three mother wavelet families – from left Daubechies (**a**), Symlet (**b**) and Coiflet (**c**).

**Figure 15:**
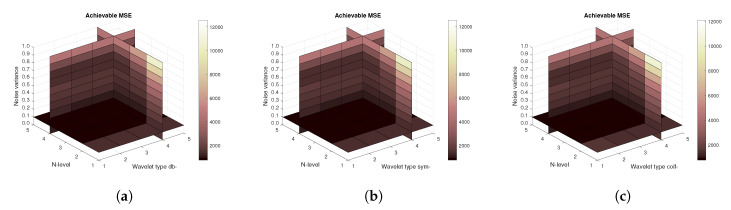
Mean achievable MSE values for 15 CT images from 5 patients with use of three mother wavelet families – from left Daubechies (**a**), Symlet (**b**) and Coiflet (**c**).

**Figure 16:**
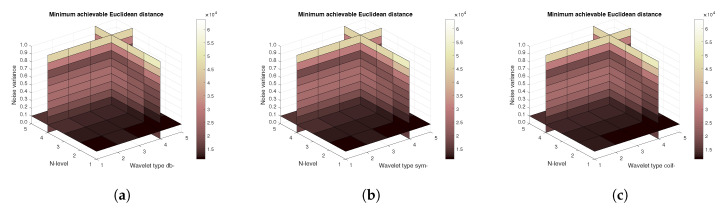
Mean achievable Euclidean distances for 15 CT images from 5 patients with use of three mother wavelet families – from left Daubechies (**a**), Symlet (**b**) and Coiflet (**c**).

Figure 14, Figure 15 and Figure 16 show a similar analysis for 15 CT scans of blood vessel calcification. The mean achievable correlation (Figure 14), mean achievable MSE values (Figure 15) and mean Euclidean distance (Figure 16) are shown, respectively. The results correspond to those achieved with the MRI scans. Again, differences among the three tested wavelet families are negligible.

**Figure 17:**
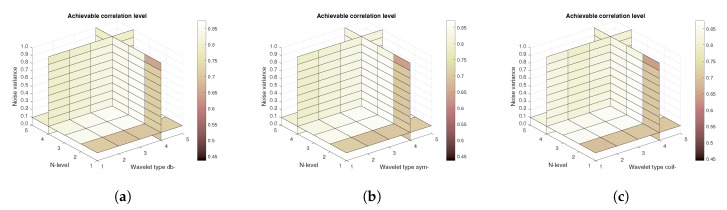
Achievable correlation levels in the presence of AWGN only for the CT scan from Figure 4 with use of three mother wavelet families—from left Daubechies (**a**), Symlet (**b**) and Coiflet (**c**).

**Figure 18:**
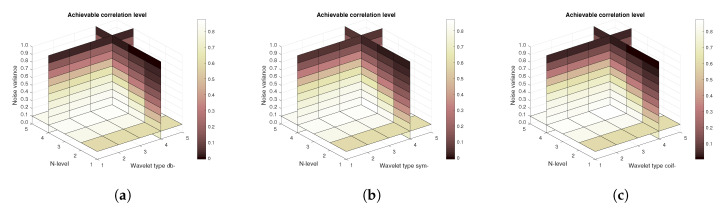
Achievable correlation levels in the presence of Salt and Pepper noise only for the CT scan from Figure 4 with use of three mother wavelet families—from left Daubechies (**a**), Symlet (**b**) and Coiflet (**c**).

In the next step, a contribution of each used noise type (AWGN and Salt and Peppers) is scrutinized. Figure 17 and Figure 18 compare the achievable correlation levels for the single image in which only a single type of noise is present. As demonstrated in Figure 17, the denoising process is highly resistant against AWGN. Whereas, as shown in Figure 18, the Salt and Pepper causes a much faster drop in achieved correlation level. Comparing the Figure 18 with the previous results, it can be concluded that the major contributor in the drop of the correlation level is specifically the Salt and Pepper type of noise.

**Figure 19:**
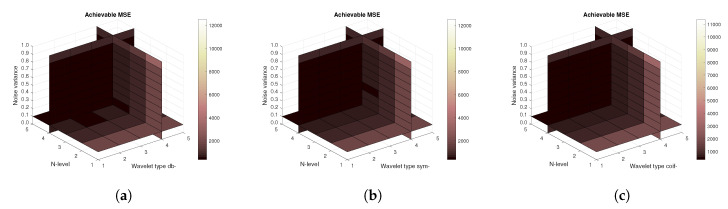
MSE in the presence of AWGN only for the CT scan from Figure 4 with use of three mother wavelet families—from left Daubechies (**a**), Symlet (**b**) and Coiflet (**c**).

**Figure 20:**
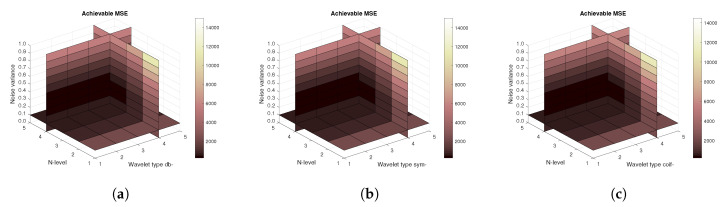
MSE in the presence of Salt and Pepper noise only for the CT scan from Figure 4 with use of three mother wavelet families—from left Daubechies (**a**), Symlet (**b**) and Coiflet (**c**).

**Figure 21:**
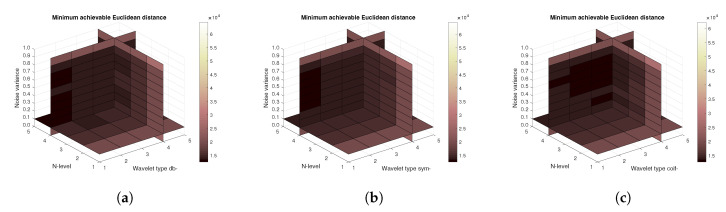
Euclidean distance in the presence of AWGN only for the CT scan from Figure 4 with use of three mother wavelet families—from left Daubechies (**a**), Symlet (**b**) and Coiflet (**c**).

**Figure 22:**
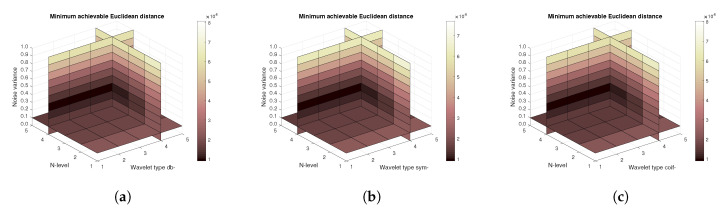
Euclidean distance in the presence of Salt and Pepper noise only for the CT scan from Figure 4 with use of three mother wavelet families—from left Daubechies (**a**), Symlet (**b**) and Coiflet (**c**).

Figure 19, Figure 20, Figure 21 and Figure 22 also compare the achieved MSE levels and Euclidean distances. From the achieved results it can be concluded that for Salt and Pepper noise there is almost no difference among the wavelet families. However, in the presence of AWGN there are observable differences. Especially with the Coiflet wavelet family, it is seen that the higher values of noise are making the denoise process performing worse.

Finally, we can return to the 1D examples of EMG signals for which a publicly available database was used from Reference [56]. The database contains recordings for six types of hand movements. Each of them has 30 recorded signals, in two channels for each of the 6 type of hand movements.

**Figure 23:**
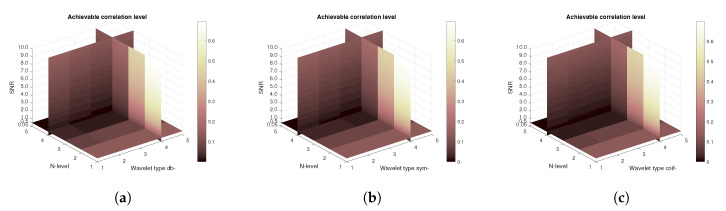
Mean achievable correlation levels of all EMG signal with use of three mother wavelet families—from left Daubechies (**a**), Symlet (**b**) and Coiflet (**c**).

**Figure 24:**
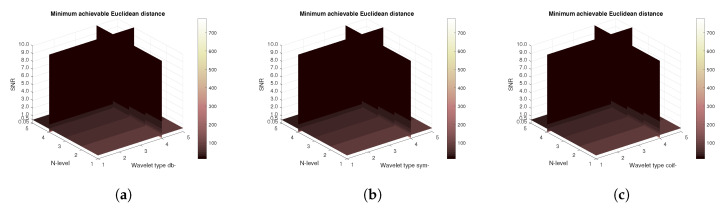
Mean achievable Euclidean distances of all EMG signal with use of three mother wavelet families—from left Daubechies (**a**), Symlet (**b**) and Coiflet (**c**).

Unlike the 2D case of MRI and CT scans in the following case the increasing z-coordinate is showing the signal’s SNR. Also, the increment in the direction of z-axis is finer than in the case of MRI and CT scans. Figure 23 are demonstrating the mean achievable correlation for all subjects. Unlike the MRI and CT scans, some differences among the families are observable. The worst performer is the Coiflet wavelet family. As the achieved MSE values are identical for all tested wavelet families the plots are not being shown. There is no significant difference among the values between families. Conversely, there are slight difference as shown on Figure 24 showing the mean Euclidean distances. Lower Euclidean distances are observed.

In the last part of the quantitative analysis, we provide the results of statistical testing. Within the statistical testing, we evaluate distribution of evaluation parameters of correlation index, MSE and Euclidean distance in the sense of normality testing. In the consecutive part, we provide a pair testing between CT and MR images for individual Wavelet families. This testing has a potential to objectively evaluate whether respective Wavelet family has statistically significant different effectivity for various datasets.

Firstly, we provide the testing of normality of evaluation parameters. We used the Chi-squared test for the normality evaluation for the Salt and Pepper and Gaussian noise (Table 1 and Table 2). The tests, where we dot reject the normality are indicated as green (α>0.05) and reject normality (α<0.05), are red. Normality tests were done on the significance level: α=0.05. For this test we define the following hypothesis:

**H_0_**: Data comes from normal distribution

**H_A_**: NOT *H*_0_

**Figure 25:**
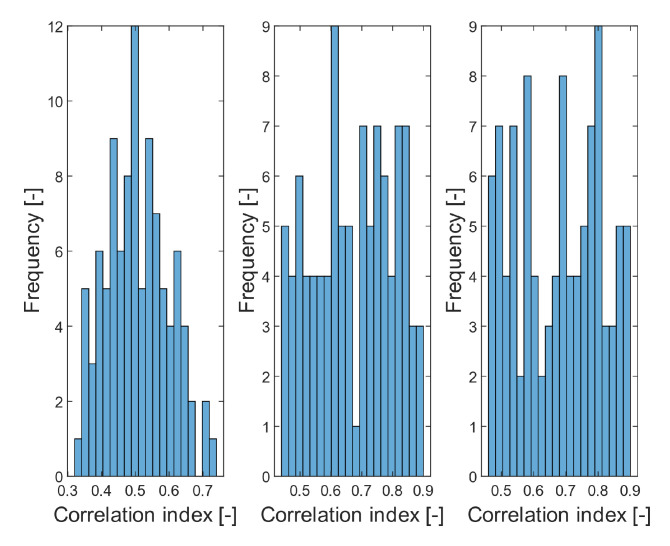
A comparison of distribution of correlation index for CT images, corrupted with Salt and Pepper noise with dynamic intensity: *d* = 0.01–0.65: Daubechies family (**left**), Symlet (**middle**) and Coiflet (**right**).

Since most of the *p*-values are less than 0.05, we generally consider the distribution of the evaluation parameters as not normally distributed. Therefore, we use Mann-Whitney statistical test for the comparison of evaluation parameters distributions. Based on the *p*-values, Daubechies wavelet distribution are mostly normally distributed, when comparing with other wavelets. Thus, we show the extract (Figure 25) of individual tested Wavelets for correlation index distributions.

After the normality evaluation, we performed the statistical testing of average value between the CT and MR images for all the Wavelets. Since we cannot generally consider all the distributions as normally distributed, we use the Mann-Whitney tests for the comparison of medians (Table 3). The aim of this comparison is the robustness evaluation for all the analyzed Wavelet families between CT and MR images. By this way, we evaluate whether a Wavelet family smoothness, effectivity is stable among different datasets. For the testing, we define the following hypothesis, where $\tilde{X_{CT}}$, $\tilde{X_{MR}}$ represent a median of respective evaluation parameter (Corr, MSE or ED) for set of CT, respective MR images.

**H_0_**: $\tilde{X_{CT}}=\tilde{X_{MR}}$

**H_A_**: NOT *H*_0_

The statistical testing based on the Mann-Whitney test indicates that Daubechies Wavelet is mostly robust in evaluation parameters for the CT and MR datasets because p-values are mostly greater than 0.05, so we do not reject the null hypothesis, except for the Euclidean distance, where we reject the null hypothesis and we can conclude that medians are not equal. This testing generally indicates that the Wavelet effectivity, measured based on the evaluation parameters generally differ for various datasets.

### 5.1. Time Consumption of Algorithm

One of the important tasks in each algorithm is a time consumption. Such parameter partially determines the method efficacy. In this section, we provide the results of time consumption for generating volumetric response of the proposed method. We provide testing on CT and MR images for Salt and Pepper (Table 4) and Gaussian (Table 5) noise. For each test, we used 30 images. We provide two tests, we performed testing on PC with the configuration (conf 1): 4-core Intel Core i5-10300H processor (2.5 GHz, TB 4.5 GHz, HyperThreading); 8 GB RAM DDR4, and these results we compare with GPU processing (conf 2) on NVIDIA GeForce RTX 2060 6 GB GDDR6. All the tests are calculated in seconds. The best results are indicated as green. Based on the experimental results, we found the less time demanding processing of images, corrupted with Gaussian noise. Among individual Wavelet families, for Salt and Pepper noise we found Symlet as the least time demanding, contrarily in the case of Gaussian noise it is Daubechies Wavelets. Generally, this experimental testing well demonstrates the effect of the possibility of GPU processing, which reduces time consumption.

### 5.2. A Subjective Evaluation of Wavelet Effectivity

The noise is basically often present in diagnostic imaging methods. Nowadays, the research has tended to focus on importance of noise reducing for example, References [59,60,61]. In connection with previous findings, reducing noise from these images is a complex task, but very important. From a clinical point of view, filtration algorithms must maintain the visibility of the important anatomical structures during noise removal. Reducing the amount of noise degradation can make the diagnosis more accurate. In Figure 4, significant suppression of anatomical areas of interest such as the right renal vein, renal arteries, colonic branches, and marked calcifications in the iliac arteries can be seen. Consequently, evaluating such an image would be almost impossible. The filtering output significantly suppresses image noise degradation while maintaining the visibility of the above-mentioned areas of interest. Sufficiently results are as well obtained from MRI of the knee (see Figure 5). Anatomical structures such as knee cartilage, lateral meniscus anterior/posterior horn, popliteus tendon and many others are more likely to be diagnosed. It seems that our method has beneficial and practical application. Especially with reducing the amount of noise degradation while preserving important anatomical structures.

## 6. Summary and Discussion

In this paper, we propose a recommendation method for modeling spatial and volumetric response of Wavelet settings for various data smoothing. Three different wavelet families (Daubechies, Symlet and Coiflet) were exploited to denoise 1D and 2D biomedical signals and images—namely EMG signals, MRI and CT scans. The proposed method is aimed to generate the spatial map of selected evaluation parameter, showing a distribution of similarity or difference of smoothed data against the gold standard via spatial Wavelet response. This method identifies more suitable Wavelet’s settings for particular application of data smoothing. The second part of the proposed method deals with the volumetric response, in which individual layers correspond with a certain level of dynamical noise influence. This method is capable of tracking dynamic features and robustness of respective Wavelet’s settings for particular data. In our analysis, we present the results of the 1D EMG signals and 2D data of musculoskeletal images, nevertheless the proposed system is generally applicable for various signals and images.

It was found out that the MR and CT scans have similar behaviour in the sense of achievable denoising effect. Generally, the chosen wavelet family, out of the three tested, has rather insignificant influence on the achieved result in both MRI and CT scans, respectively. The major contributor in the image degradation is Salt and Pepper noise. At some level of added Salt and Pepper noise all three wavelet families are starting to be identically ineffective. Our analysis offers a comparison of three various Wavelet families. In order to identify statistically significant differences among individual Wavelet settings, we also present the statistical testing based on Mann-Whitney test, comparing medians of the response for Gaussian and Salt and Pepper noise. Based on this testing, Daubechies Wavelet appears to be mostly robust in evaluation parameters, except for Euclidean distance. One of the significant attributes of this algorithm is time consumption, showing a certain view on the method’s effectivity. We compared computing on processor with GPU processing for Salt and Pepper and Gaussian noise. While for the Salt and Pepper noise we evaluated Symlet as the most effective, for Gaussian noise Daubechies appear as the most effective. The experimental results also point out on the fact that GPU processing reaches better results from the view of time consumption.

The proposed method shows a distribution of selected evaluation parameters. Thus, this method can predict more suitable Wavelet settings and its robustness for particular application. One of the limitations can be classification of a specific Wavelet. Adding a classification procedure, which would autonomously recognize the most suitable Wavelet would contribute to performance of the proposed method. Since we simultaneously process a big amount of data (types of Wavelets and decomposition levels), we should be aware of higher time consumption requirements. On the other hand, GPU processing enables better performance with reduced time consumption, which optimizes method’s effectivity. We should also mention that the evaluation system is based on the comparison against the gold standard images as a reference, which may be limitation for some application. Another possible source of limitation is that the data was provided from public databases. However, these data were used for initial testing of the proposed methodology, which was fully sufficient. We are currently working on testing on self-measured data. In the contrast with the aforementioned limitations, the proposed method significantly contributes to Wavelet evaluation via multiple Wavelet response with the potential of simulate Wavelet response. Furthermore, the proposed system is versatile and can be used for any form of signals and images.

## 7. Conclusions

This paper presents the aspects of the spatial and volumetric modelling of the Wavelet family response for three tested Wavelet families. Such approaches allow for analyzing and evaluating the most appropriate Wavelets from respective family to be used for data smoothing. Also, volumetric modelling enables tracking the dynamical features of selected Wavelets upon the dynamical effect. The proposed method generates the spatial map of respective evaluation parameter distribution, showing the similarity or difference of respective Wavelet settings against the gold standard images. In our analysis, we use correlation index as a parameter of similarity, MSE and ED as difference parameters. The second part of the proposed system enables tracking and extraction features of individual Wavelets when dynamical noise is present. This approach allows for the simulation of the robustness of respective settings within the data degradation.

In our analysis, we provide testing of the proposed spatial and volumetric response on CT and MR blood vessels and musculoskeletal images, as well as 1D EMG signals. In order to test robustness of individual Wavelet settings, we bring a comparative analysis of additive Gaussian and Salt and Pepper noise to evaluate differences in Wavelet response. In this context, we provide the statistical analysis of statistical testing of the Wavelet response for individual noise models between CT and MR images. Since we cannot ensure the data normality based on the Chi-squared test, we use Mann-Whitney tests to evaluate differences for individual Wavelet families. The statistical testing based on the Mann-Whitney tests indicates that the Daubechies Wavelet is mostly robust in evaluation parameters for the CT and MR datasets because the p-values are mostly greater than 0.05, except for the Euclidean distance. We also provide an analysis of time consumption, representing time demands for generating a Wavelet response. Based on the experimental results it seems that GPU processing allows for the reduction of time consumption. We also note differences in the time consumption between Salt and Pepper and Gaussian noise. In this context, the application of Gaussian noise enables the reduction of time consumption.

The proposed model enables the computing of simultaneous Wavelet features for various Wavelets and its decomposition levels via spatial and volumetric responsive models. Besides certain limitations such is time consumption or evaluation against gold standard, the proposed method brings valuable simultaneous prediction for multiple Wavelet settings, which predicts the Wavelet efficacy and robustness. In the future time, it would be worth extending the proposed method about the classification procedure based on a matrix decomposition. Such improvement would enable classify a finite range of the most suitable Wavelet settings from others based on objective parameters. This would make a completely autonomous system for the most Wavelet settings recommendation.

## Figures and Tables

**Table 1:** Distribution of p-values of Chi-squared test normality for the Salt and Pepper noise with dynamical noise intensity: *d* = 0.001–0.65.

Wavelet [Corr|MSE|ED]	CT Images			MR Images		
Daubechies	0.12	0.21	0.18	0.02	0.41	0.21
Symlet	0.03	0.01	0.01	0.04	0.01	0.26
Coiflet	0.21	0.04	0.03	0.01	0.23	0.25

**Table 2:** Distribution of p-values of Chi-squared test normality for the Gaussian noise with dynamical noise intensity: μ=0,σ = 0.01–0.6.

Wavelet [Corr|MSE|ED]	CT Images			MR Images		
Daubechies	0.09	0.12	0.07	0.04	0.35	0.04
Symlet	0.08	0.11	0.03	0.01	0.11	0.31
Coiflet	0.14	0.08	0.04	0.02	0.17	0.17

**Table 3:** Distribution of p-values of Mann-Whitney test for the Gaussian and Salt and Pepper noise.

Wavelet [Corr|MSE|ED]	CT Images			MR Images		
Daubechies	0.65	0.78	0.03	0.48	0.64	0.02
Symlet	0.04	0.70	0.02	0.02	0.25	0.01
Coiflet	0.01	0.01	0.01	0.01	0.01	0.01

**Table 4:** A comparison of time consumption (calculated in seconds) for Salt and Pepper noise for CT and MR images and individual Wavelet families.

Wavelet Type	CT Images|conf 1|conf 2|		MR Images|conf 1|conf 2|	
Daubechies	1320	941	1281	845
Symlet	1148	847	1041	621
Coiflet	1245	912	1141	697

**Table 5:** A comparison of time consumption (calculated in seconds) for Gaussian noise for CT and MR images and individual Wavelet families.

Wavelet Type	CT Images|conf 1|conf 2|		MR Images|conf 1|conf 2|	
Daubechies	945	516	987	541
Symlet	969	547	1011	654
Coiflet	1102	874	989	555

## Abbreviations

The following abbreviations are used in this manuscript:

CT	Computed tomography
MR	Magnetic resonanceEMG
EMG	Electromyography
MSE	Mean Squared Error
ED	Euclidean distance
Corr	Correlation index
AWGN	Additive White Gaussian Noise
NLM filter	Non Local Mean filter
MRF	Markov Random Field
BLS-GSM	Bayes Last Square Gaussian Scale Mixture
FT	Fourier transform
FFT	Fast Fourier transform
DCT	Discrete cosine transform
ECG	Electrocardiography
DWT	Discrete Wavelet Transform
PSNR	Peak Signal-to-Noise Ratio
SNR	Signal-to-Noise
